# Differences in PGE_2_ Production between Primary Human Monocytes and Differentiated Macrophages: Role of IL-1β and TRIF/IRF3

**DOI:** 10.1371/journal.pone.0098517

**Published:** 2014-05-28

**Authors:** Yukinori Endo, Ksenia Blinova, Tatiana Romantseva, Hana Golding, Marina Zaitseva

**Affiliations:** Center for Biologics Evaluation and Research (CBER), Food and Drug Administration (FDA), Bethesda, Maryland, United States of America; University of California Merced, United States of America

## Abstract

Prostaglandin E2 (PGE_2_) is induced *in vivo* by bacterial products including TLR agonists. To determine whether PGE_2_ is induced directly or via IL-1β, human monocytes and macrophages were cultured with LPS or with Pam3CSK4 in presence of caspase-1 inhibitor, ZVAD, or IL-1R antagonist, Kineret. TLR agonists induced PGE_2_ in macrophages exclusively via IL-1β-independent mechanisms. In contrast, ZVAD and Kineret reduced PGE_2_ production in LPS-treated (but not in Pam3CSK4-treated) monocytes, by 30–60%. Recombinant human IL-1β augmented *COX-2* and *mPGES-1* mRNA and PGE_2_ production in LPS-pretreated monocytes but not in un-primed or Pam3CSK4-primed monocytes. This difference was explained by the finding that LPS but not Pam3CSK4 induced phosphorylation of IRF3 in monocytes suggesting activation of the TRIF signaling pathway. Knocking down *TRIF*, *TRAM*, or *IRF3* genes by siRNA inhibited IL-1β-induced *COX-2* and *mPGES-1* mRNA. Blocking of TLR4 endocytosis during LPS priming prevented the increase in PGE_2_ production by exogenous IL-1β. Our data showed that TLR2 agonists induce PGE_2_ in monocytes independently from IL-1β. In the case of TLR4, IL-1β augments PGE_2_ production in LPS-primed monocytes (but not in macrophages) through a mechanism that requires TLR4 internalization and activation of the TRIF/IRF3 pathway. These findings suggest a key role for blood monocytes in the rapid onset of fever in animals and humans exposed to bacterial products and some novel adjuvants.

## Introduction

Fever is a homeostatic response of the host to the infection by microbial and viral pathogens. The invading pathogens are sensed by Pattern Recognition Receptors (PRR) including Toll-like receptors (TLR) expressed by the cells at the site of the infection. The resulting local inflammatory response culminates in release of pyrogenic cytokines such as IL-1β, TNF-α, and IL-6. According to the conventional view of the mechanism of fever, locally-induced pyrogenic cytokines are transported by the blood stream to the “fever producing center”, the ventromedial preoptic area (VMPO) of the anterior hypothalamus, where they activate generation and release of the prostaglandin (PG)E_2_, a thermogenic lipid mediator [1]. This concept of cytokine-induced fever was challenged by studies showing that neither LPS nor IL-1β administered peripherally crossed the blood-brain barrier and that LPS injected intravenously in animals induced febrile responses and PGE_2_ in VMPO before cytokines were elevated in the blood [2], [3]. Studies in mice genetically engineered to lack pyrogenic cytokines, the ability of PGE_2_ to cross blood-brain barrier, and observations of clinical fevers that frequently occur without increase of circulating cytokines suggested alternative routes for transmission of febrile signals [3]–[7].

According to this view, activation of PRR in tissue macrophages triggers production of PGE_2_ that can transmit fever signal from infected tissue via the blood stream or via the neural route (e.g., vagus nerve) [8]. Studies in rats demonstrated high levels of cyclooxygenase (COX)-2 protein (the major enzyme in PGE_2_ biosynthesis) in hepatic and pulmonary macrophages but not in the brain tissue following administration of LPS [9]. Furthermore, depletion of macrophages from peripheral tissues resulted in attenuation of LPS-induced fever in rats and in guinea pigs [10], [11]. The *in vivo* effects of LPS on tissue macrophages were confirmed showing that LPS induced PGE_2_ production by isolated Kupffer cells in guinea pigs and rats [12], [13]. It is important to note that Kupffer cells are activated by pathogens and microbial products in the circulation. Pathogens that invade tissues are recognized by inflammatory monocytes that are recruited from the blood stream to the site of the infection. Recent studies from our laboratory showed that in addition to macrophages, primary human monocytes produce high quantities of PGE_2_ and of IL-1β following *in vitro* stimulation with several TLR agonists [14]. However, it was not clear whether microbial products induce PGE_2_ in macrophages and monocytes directly or it is triggered by IL-1β produced at the site of infection.

In addition to LPS, other TLR agonists have been shown to induce fever in animal models: the TLR2 agonists, macrophage-activating lipopeptide-2 (MALP-2) from *Mycoplasma fermentans* (*M. fermentans*), and a synthetic analogue of fibroblast-stimulating lipopetide-1 (FSL-1) spanning the NH_2_-termial sequence of a lipoprotein from *M. salivarium*, as well as TLR9 agonists synthetic oligonucleotide (ODN) of bacterial DNA CpG [15]–[17]. Importantly, several studies (including in our laboratory) showed that TLR2, TLR5, and TLR9 agonists up-regulated PGE_2_ *in vivo* [14], [16], [17]. While TLR2 and TLR9 agonists signal exclusively through adaptor molecule MyD88, TLR4 signaling may proceed via either MyD88 or through TRIF resulting in activation of interferon regulatory factor (IRF)-3 and a subsequent induction of the type 1 interferon (IFN-β) [18]. However, whether TLR4-TRIF signaling plays a role in PGE_2_ production has not been addressed yet.

In the present study, we assessed the role of IL-1β in the production of PGE_2_ by human monocytes and differentiated macrophages activated with the TLR4 agonist LPS or with TLR2 agonists FSL-1 and Pam3CSK4. Our data demonstrated that human macrophages and monocytes engage different signaling pathways leading to PGE_2_ production. Differentiated macrophages use an IL-1β-independent pathway exclusively. In contrast, in monocytes PGE_2_ is up-regulated both by IL-1β-dependent and IL-1β-independent pathways. The IL-1β-dependent pathway is restricted to TLR4 (but not TLR2) agonists and involves TRIF/IRF3 signaling.

## Methods

### Reagents

The following reagents were used in the study: highly purified LPS (LPS *E. coli*, Serotype O111:B4,TLR*grade*™, Enzo Life Sciences, Inc., Farmingdale, NY) synthetic bacterial lipoprotein, Pam3CSK4 (TLR1/2 ligand), a synthetic diacylated lipoprotein FSL-1 (TLR2/6 ligand), all from InvivoGen (San Diego, CA); ZVAD (Z-Tyr-Val-Ala-Dl-Asp-Fluoromethylketone, Bachem Americas, Inc., Torrance, CA), Kineret (Amgen, Thousand Oaks, CA), human recombinant IL-1β (rIL-1β, Endotoxin level <0.1 ng/µg, Life Technologies, Grand Island, NY), and dynasore (Enzo).

### Cells, cell culture, and measurements of IL-1β, IL-6 and of PGE_2_

Human elutriated monocytes from healthy donors were isolated using counterflow centrifugal elutriation and were obtained from the Department of Transfusion at the National Institutes of Health (Bethesda, MD). Macrophages were differentiated in macrophage serum-free medium (Life Technologies) in the presence of 10 ng/ml of human recombinant GM-CSF (PeproTech Inc., Rocky Hill, NJ) for 5–6 days at 37°C and 5% CO_2_.

Human elutriated monocytes and differentiated macrophages were cultured in triplicates at 1–1.5×10^6^ cells/ml alone or with TLR agonists in the presence or absence of ZVAD at 10 µg/ml (20 µM) or Kineret at 100 µg/ml in RPMI medium supplemented with 2% human heat-inactivated AB serum (Mediatech, Manassas, VA) or with 15% of Fetal Bovine Serum (FBS) for cytokines and PGE_2_, respectively.

In some experiments, human elutriated monocytes were primed with LPS or with Pam3CSK4 for 1 h followed by overnight incubation with rIL-1β at 100 ng/ml. At the end of cell culture period, cells and cell culture supernatants were harvested to assess expression of *COX-2* and *mPGES-1* mRNA and of PGE_2_ production, respectively.

In some experiments, monocytes were treated with GTPase inhibitor dynasore at 10 µM for 1 h prior to priming with LPS for 1 h and subsequent incubation with 100 ng/ml of human rIL-1β for 18 h. At the end of the incubation period, cell culture supernatants were assayed for PGE_2_ production.

IL-1β and IL-6 were measured in the cell culture supernatants using human Quantikine ELISA kits (R& D Systems, Minneapolis, MN) and Synergy2 Multi-Mode (BioTek Instruments, Winooski, VT); PGE_2_ was detected using PGE_2_ Homogeneous Time-Resolved Fluorescence assay (HTRF) Kit (Cisbio Bioassays, Bedford, MA) and Novostar plate reader (BMG Labtech, Offenburg, Germany). PGE_2_ concentrations were calculated using four-parameter logistic fit using Origin software application (Origin Lab, Northampton, MA). The detectable PGE_2_ range was from 10 to 5000 pg/ml.

The study received an exempt status by the RIHS Committee at CBER, FDA; protocol 03-120B.

### qPCR for *COX-2* and *mPGES-1* mRNA expression

Cells were subjected to lysis in RLT buffer (RNeasy, QIAGEN, Frederick, MD), homogenized with QIAshredder (QIAGEN), and snap frozen on dry ice. Total RNA was isolated according to the manufacturer's protocol. cDNAs were obtained using Reverse transcriptase SuperScript VILO (Life Technologies) according to the manufacturer's instructions. qPCR was performed using Power SYBR green (Applied Biosystems, Carlsbad, CA) and a 7900HT Fast Real-Time PCR system (Applied Biosystems). The following primer pairs were used: *COX-2* (sense: 5′-GAATCATTCACCAGGCAAATTG-3′ and antisense: 5′-TTTCTGTACTGCGGGTGGAAC-3′), *mPGES-1* (sense: 5′-CTGCTGGTCATCAAGATGTACG-3′ and antisense: 5′-GGTTAGGACCCAGAAAGGAGT-3′), and *β-actin* (sense: 5′-CCTCACCCTGAAGTACCCCA-3′ and antisense: 5′-TGCCAGATTTTCTCCATGTCG-3′). The cycling conditions were as follows: 95°C for 10 min followed by 45 cycles of 95°C for 15 s, 60°C for 1 min and 95°C for 15 s. Fluorescence thresholds (C_t_) were determined automatically by software, with efficiencies of amplification for the studied genes ranging between 92 and 110%. The ΔC_t_ value for each cDNA sample was calculated by subtracting C_t_ value of the reference gene *β-actin* from the C_t_ value of the target sequence. Fold increase of mRNA expression was calculated following manufacturer's instructions and using standard formula, Fold increase  = 2^−ΔΔCt^.

### Western Blotting

#### COX-2

Cells were lysed in 1% NP40 lysis buffer with Protease Inhibitors (PI) and PMSF on ice for 30 min. Cell lysates from macrophages and monocytes were resolved using 4–20% gradient gels (Pierce, Rockford, IL) and Hepes SDS running buffer.

#### IRF3

Monocytes or macrophages were primed with 1 ng/ml of LPS or with 50 ng/ml of Pam3CSK4 for 1 h. At the end of incubation period, cells were lysed in 1% NP40 lysis buffer with PI, PMSF, and PhosSTOP Phosphatase Inhibitor (Roche Diagnostics, Indianapolis, IN) on ice for 30 min. Cell lysates were resolved using 7.5% Tris-Glycine Page Gold Precast gel (Lonza, Rockland, ME) and Tris-Glycine SDS running buffer.

#### NF-κB

Nuclear extracts were prepared from monocytes as previously described [19]. In brief, harvested and pelleted monocytes were incubated in 400 µl of cold buffer A (10 mM Hepes pH 7.9; 10 mM KCl; 0.1 mM EDTA; 10 mM EGTA; 1 mM DTT and PI) on ice for 15 min, followed by addition of 25 µl of 10% NP40 followed by vortexing and centrifugation at 15,000 rpm for 1 min. The supernatant was removed and nuclear pellet was re-suspended in ice cold buffer C (20 mM Hepes pH 7.9; 0.4 M NaCl; 1 mM EDTA; 1 mM EGTA; 1 mM DTT, and PI), incubated on ice with vigorous shaking for 15 min, and centrifuged at 15,000 rpm for 1 min. The supernatant was collected and proteins were resolved using 7.5% Tris-Glycin extended (TGX) precast gel (Bio-Rad, Laboratories, Hercules, CA).

In all experiments, following SDS-PAGE, the proteins were transferred to PVDF membranes and probed in the Western Blotting with antibodies: rabbit polyclonal anti-COX-2 Ab (Cat ab52237, Abcam, Cambridge, MA); rabbit anti-IRF3 mAb (Cat 4302) and rabbit anti- IRF3-P mAb (Ser 396, Cat 4947), both from Cell Signaling (Danvers, MA); rabbit anti-NF-κB p65 Ab (Cat 3987); anti-β-actin mAb (Cat ab20272) and anti-TATA binding protein (TBP) Ab (Cat ab818), both from Abcam. Donkey HRP-anti-rabbit and sheep HRP-anti-mouse Abs were used as secondary reagents, both from GE Healthcare Bio-Sciences Corp. (Piscataway, NJ). Densitometry was performed using AlphaImager 3400 Imaging System (Alpha Innotech Corp., San Leanrdo, CA).

### RNA Interference

Human astrocytoma U373-CD14 [20] was kindly provided by Dr. Katherine Fitzgerald [21] and maintained in 10% fetal bovine serum (FBS) DMEM medium with 100 U/ml penicillin and 100 µg/ml streptomycin as previously described [22].

Negative control siRNA was purchased from QIAGEN. siRNA targeting *TRIF (TICAM1)*, *TRAM (TICAM2)* and *IRF3* were from Thermo Scientific (Lafayette, CO). The following target sequences of siRNAs were used: *TRIF* (GGAGCCACAUGUCAUUUGG, CCAUAGACCACUCAGCUUU, GGACGAACACUCCCAGAUC, and CCACUGGCCUCCCUGAUAC); *TRAM* (GAAGAUCUAUCCUUGUGUA, UGGACAAUCUUAUUACUGA, UAAGAGAUACUUGGUGUAA, and CCUCAAAUUUGUGAUAUUG); *IRF3* (CGAGGCCACUGGUGCAUAU, CCAGACACCUCUCCGGACA, GGAGUGAUGAGCUACGUGA, and AGACAUUCUGGAUGAGUUA).

One day before the experiment, U373-CD14 cells were seeded at 4×10^4^ cells/well in antibiotic-free DMEM medium containing 10% FBS (DMEM/FBS) in 24 well-plate (Corning). Immediately before siRNA transfection, the medium was changed to serum-free antibiotic-free DMEM. U373-CD14 cells were transfected with siRNAs at 200 nM using Lipofectamine 2000 (Life Technologies) for 5–7 h followed by culture in fresh DMEM/FBS for 48 h.

Efficiency of gene knock-down by siRNA was confirmed using qPCR and Western Blotting. For the qPCR, the following primers were used: IRF3 cat. No: QT00012866; TRIF (TICAM1) cat. No: QT00201614; TRAM (TICAM2) cat. No: QT02407048 purchased from QIAGEN (www.qiagen.com/GeneGlobe). For the Western Blotting of the cell lysates prepared from transfected cells and resolved in SDS-PAGE, the following antibodies were used: goat anti-human TRIF Ab (Cat AF6216, R&D Systems), rabbit anti-human TRAM Ab (Cat ab17221, Abcam), and rabbit anti-human IRF3 Ab (Cat 4302, Cell Signaling). Donkey HRP-anti-rabbit (GE Healthcare) and bovine HRP-anti-goat (Jackson Immune Research Laboratories, Inc., West Grove, PA) antibodies were used as secondary reagents (WB, lower panels in A, B, and C, respectively). The experiment was performed 3 times with similar results.

On the day of the experiment, transfected U373-CD14 cells were primed with 1 ng/ml LPS for 1 h and then were incubated with human rIL-1β at 100 ng/ml for additional 18 h. Cells were collected and used to assay *COX-2* mRNA expression by qPCR.

### Flow Cytometry Analysis (FACS) of IL-1R1 expression

Human elutriated monocytes or differentiated macrophages were cultured with LPS at 20 ng/ml, or with Pam3CSK4, or FSL-1 at 500 ng/ml overnight. Cells were stained with goat polyclonal anti-human IL-1R1-Phycoerythrin (PE) antibodies (cat FAB269P, R&D systems Inc., Minneapolis, MN) or with normal goat IgG-PE isotype control (cat sc-3992, Santa Cruz, Dallas, TX) in presence of human FcR blocking reagent following the manufacturer's instructions (cat 130-059-901, Miltenyi Biotech, San Diego, CA). Cells were analyzed using a FACSCalibur flow cytometer (BD Biosciences, San Jose, CA) and FlowJo 7.6.5 software (TreeStar Inc., Ashland, OR).

### Mouse experiments

Female NOD.129S2(B6)-Casp1tm1Sesh/LTJ (caspase-1 KO) mice and NOD/ShiLtJ wild type (w/t) (Jackson Laboratories, Bar Harbor, ME) were used at 8 weeks of age. Macrophages were harvested from the peritoneal cavity 3 days after intraperitoneal injection of thioglycollate solution. Mouse macrophages were incubated with LPS overnight and IL-1β and PGE_2_ were measured in the cell culture supernatant using Quantikine Mouse IL-1β Immunoassay Kit (R&D Systems Inc.) and PGE_2_ High Sensitivity EIA Kit (Assay Designs, Farmingdale, NY), respectively. The handling of mice and experimental procedures were approved by the CBER animal study review committee.

### Statistical analysis

Means were compared by paired t tests using Excel software. Data are expressed and plotted as means ± STDEV values. Sample sizes for each experimental condition are provided in the figure legends.

## Results

### Effect of caspase-1 inhibitor ZVAD on PGE_2_ production in primary human monocytes and macrophages activated with LPS

**Figure 1:**
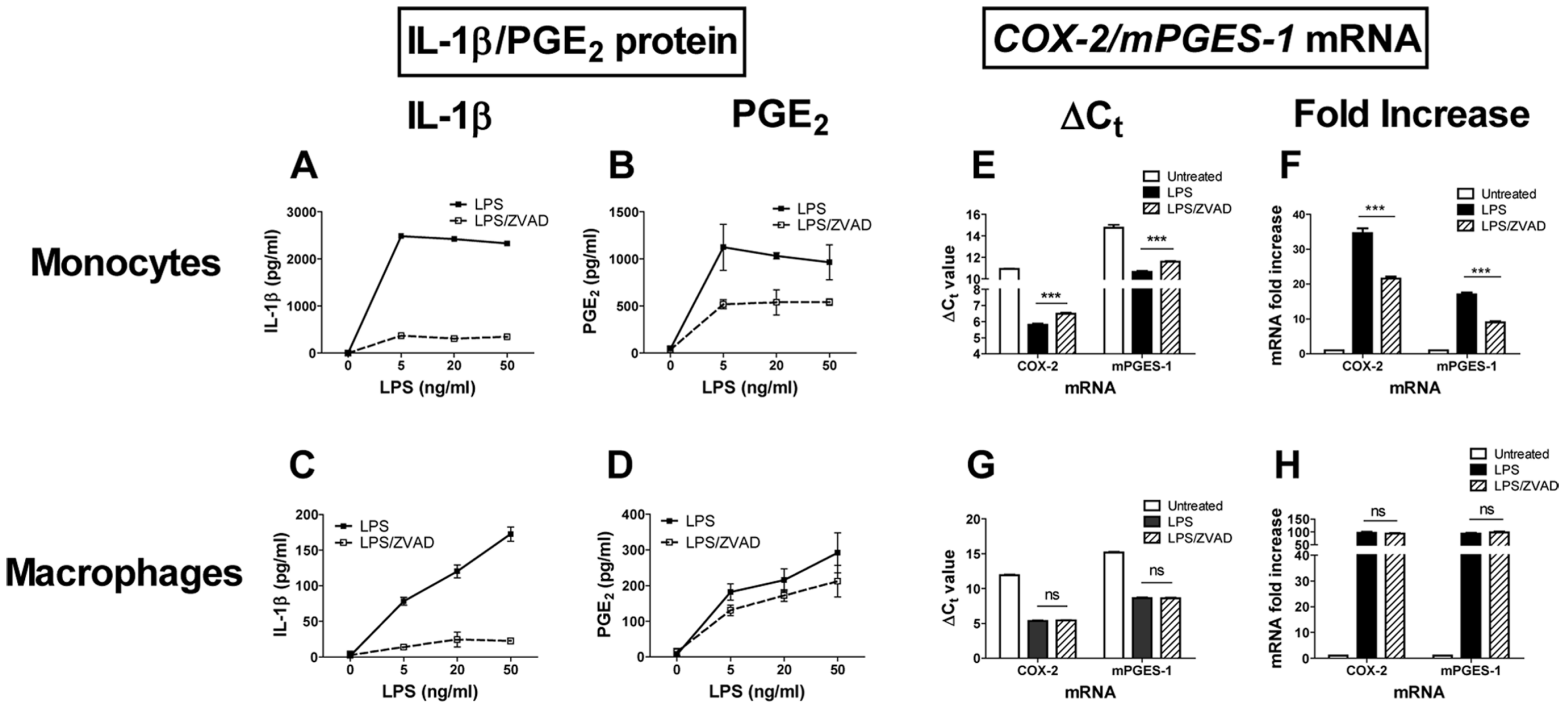
ZVAD reduced PGE_2_ production and *COX-2*/*mPGES-1* mRNA expression in LPS-activated monocytes but not macrophages. Monocytes (A, B, E, F) and differentiated macrophages (C, D, G, H) were cultured with LPS at indicated concentrations in the absence (solid lines in A–D) or in the presence of ZVAD (A–D, broken lines). After overnight incubation, cell culture supernatants were assayed for IL-1β (A, C) and for PGE_2_ (B, D). The data is shown as mean ± STDEV for PGE_2_ and IL-1β protein concentration calculated for triplicate wells. Monocytes (E, F) and macrophages (G, H) were incubated alone (open bars) or with 20 ng/ml of LPS in the absence (solid bars) or in the presence of ZVAD (striped bars) and were assayed for *COX-2* mRNA and for *mPGES-1* mRNA expression at 6 and 12 h post treatment by qPCR in triplicate wells. The C_t_ values for *COX-2* and *mPGES-1* mRNA expression in monocytes (E) and macrophages (G) were normalized using qPCR reactions with *β-actin* primers performed in the same samples and are shown as mean ΔC_t_±STDEV calculated for triplicate wells. Asterisks denote statistical significance of the differences in the means of ΔC_t_ values between LPS-treated cells cultured with or without ZVAD. ΔC_t_ values were used to calculate *COX-2* and *mPGES-1* mRNA fold increases over time 0 in monocytes (F) and macrophages (H), ***p≤0.001; ns, not significant (p≥0.05). The experiment described in Figure 1 was performed 3 times with cells obtained from different donors. The data is shown for one representative donor.

Earlier studies from our laboratory showed that human monocytes produced high quantities of both PGE_2_ and IL-1β upon *in vitro* stimulation with several TLR agonists [14]. To determine whether TLR agonists directly activate production of PGE_2_ or through IL-1β, human elutriated monocytes and differentiated macrophages were cultured *in vitro* with LPS in the absence or presence of the caspase-1 inhibitor, ZVAD. The levels of secreted IL-1β and PGE_2_ were assayed in the cell culture supernatants after 18 h of incubation (Fig. 1 A, C, and B, D, respectively). LPS induced production of IL-1β in both cell types with monocytes producing higher levels (2–3 µg/ml) than macrophages (100–200 ng/ml) (Fig. 1 A and C, solid lines). The dose response curves were also different; plateau levels of both IL-1β and PGE_2_ in monocytes were reached at 5 ng/ml and in macrophages at ≥50 ng/ml LPS. As expected, ZVAD dramatically reduced secreted IL-1β in both cell types (Fig. 1 A and C, broken lines). In contrast to IL-1β production, ZVAD did not affect LPS-induced PGE_2_ production in macrophages (Fig. 1D, broken line). At the same time, ZVAD inhibited PGE_2_ production by 51.6±3.9%, 47.3±0.38%, and 40.0±7.8% in LPS-treated monocytes derived from three donors. Representative data from one donor are shown in Fig. 1B (broken line).

COX-2 and microsomal prostaglandin (PG) E synthase enzymes (mPGES-1) play a major role in the biosynthesis of PGE_2_ (reviewed in [23]). To determine whether reduced PGE_2_ production in the presence of caspase-1 inhibitor was due to the reduced transcription of *COX-2* and/or *mPGES-1* genes, monocytes and macrophages were cultured with LPS in the absence or presence of ZVAD and assayed for *COX-2* and *mPGES-1* mRNA expressions at 6 and 12 h post treatment (peak of LPS-induced *COX-2* and *mPGES-1* mRNA up-regulation, data not shown) by qPCR (Fig. 1 E, and G). In agreement with the data in panel D, the changes in *COX-2* and *mPGES-1* ΔC_t_ values after LPS treatment of macrophages were not affected by ZVAD (no change in the C_t_ values) (Fig. 1G). However, in monocytes, *COX-2* and *mPGES-1* ΔC_t_ values were significantly higher in monocytes treated with LPS and ZVAD compared with LPS alone, indicating reduced levels of mRNA present (p≤0.001) (Fig. 1E). Calculated fold increases in *COX-2* and *mPGES-1* mRNA expression based on ΔC_t_ values confirmed that up-regulation of *COX-2* and *mPGES-1* mRNA expressions induced by LPS in macrophages were not affected by ZVAD, while in monocytes, LPS-induced increase in both genes was reduced up to 50% by ZVAD compared to LPS alone (Fig. 1F and H).

**Figure 2:**
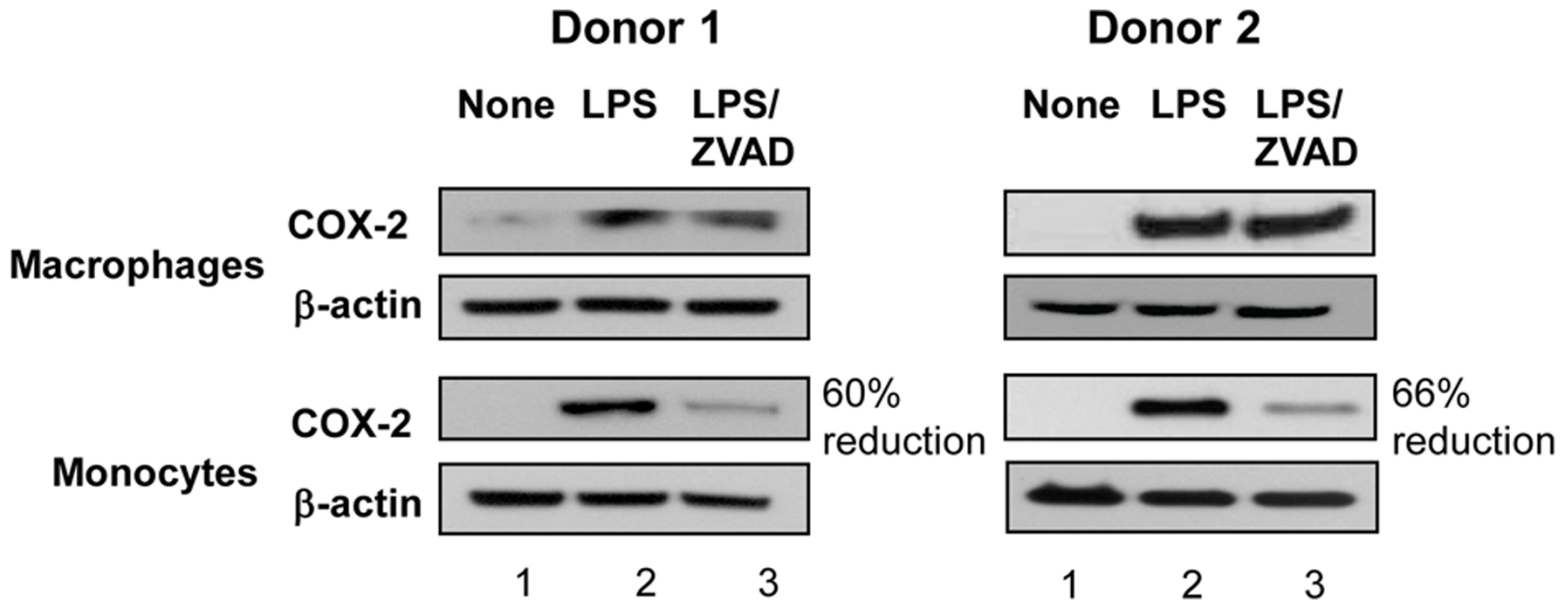
ZVAD reduced COX-2 protein expression in monocytes but not in macrophages. Macrophages and monocytes were obtained from Donor 1 and Donor 2 and were left untreated (lane 1) or were incubated with LPS at 20 ng/ml in the absence (lane 2) or in presence of ZVAD (lane 3) for 12 h. Cells were lysed in 1% NP40 and proteins were analyzed by SDS-PAGE (12 and 16 µg protein per lane for monocytes and macrophages, respectively) and Western Blotting with Abs against COX-2 (panels 1 and 3 from the top) or with β-actin as loading control (panels 2 and 4 from the top). Densitometry was performed on developed films and percent reduction of COX-2 protein expression in LPS-treated monocytes in the presence of ZVAD was calculated relative to the expression of COX-2 in monocytes treated with LPS alone.

To confirm the differential effect of ZVAD on COX-2 protein expression, cell lysates were prepared from monocytes and macrophages from two donors and were used for protein SDS-PAGE followed by Western Blotting with antibodies against COX-2 protein (Fig. 2). LPS induced an increase in COX-2 protein expression in both cell types. ZVAD treatment reduced COX-2 protein expression by 60% and 66% in Donor 1 and 2, respectively in LPS-treated monocytes but not in macrophages. These data confirmed that, unlike differentiated macrophages, COX-2 protein expression and PGE_2_ production in human monocytes partially depend on caspase-1.

### Macrophages from caspase-1 KO mice produced PGE_2_ but not IL-1β in response to LPS stimulation

**Figure 3:**
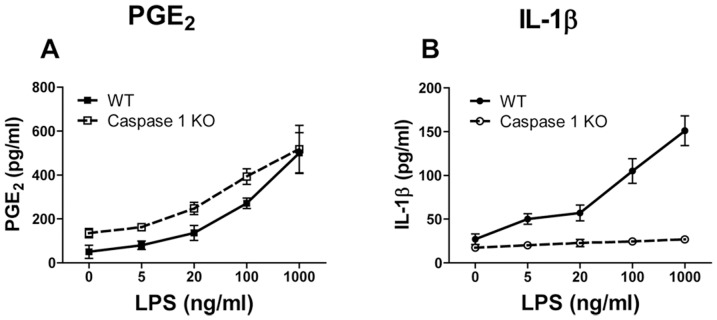
LPS induced PGE_2_ but not IL-1β secretion in macrophages from caspase-1 KO mice. Macrophages were isolated from the peritoneal cavity of wild type (WT, solid lines) or caspase-1 KO mice (broken lines) and were incubated *in vitro* with LPS for 24 h. Cell culture supernatants were assayed for PGE_2_ (A) and for IL-1β (B). The data are shown as means ± STDEV for triplicate wells. The experiment was performed three times with similar results.

To confirm the IL-1β-independent mechanism of PGE_2_ induction in macrophages in response to TLR4 activation, macrophages were isolated from the peritoneal cavity of caspase-1 KO mice and from wild type control mice and were activated *in vitro* with LPS (Fig. 3). Following 18 h incubation, cell culture supernatants were assayed for IL-1β and PGE_2_. Similar levels of PGE_2_ were produced by peritoneal macrophages from wild type and caspase-1 KO mice activated with LPS in a dose-dependent fashion (Fig. 3A). In the same cultures, only macrophages from wild type mice but not caspase-1 KO mice produced IL-1β (Fig. 3B). These results confirmed that PGE_2_ production in differentiated macrophages of both human and mouse origin is independent of caspase-1 and production of mature IL-1β.

These data suggested that two signaling pathways might be involved in the induction of PGE_2_ following LPS/TLR4 activation in cells of the monocytic lineage: caspase-1-dependent and caspase-1-independent. Both pathways are activated by LPS in monocytes, while in macrophages (human and mouse), PGE_2_ induction by LPS appeared to be caspase-1-independent.

### PGE_2_ production in monocytes in response to human recombinant IL-1β (rIL-1β) requires LPS priming

**Figure 4:**
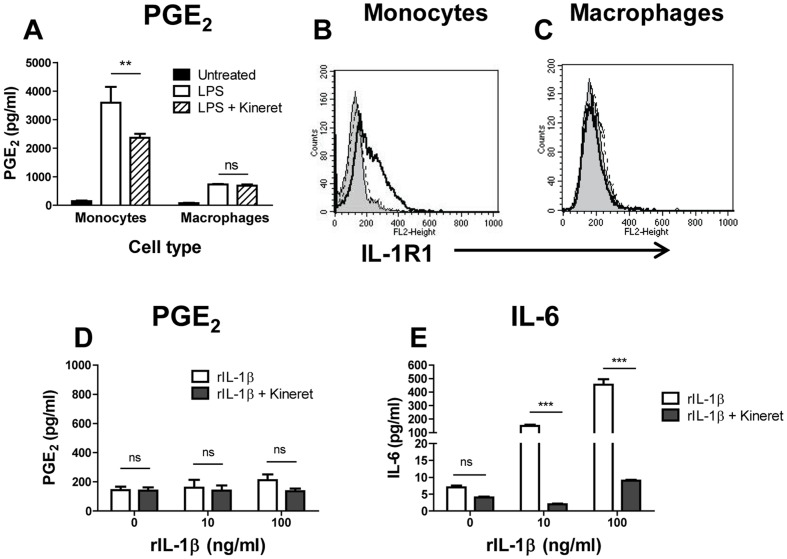
Different contributions of rIL-1β IL-1R signaling to IL-6 vs. PGE_2_ production in human monocytes and macrophages. (A) Monocytes and differentiated macrophages were left untreated (solid bars) or were incubated with LPS alone at 20 ng/ml or with LPS in the presence of the IL-1R inhibitor Kineret (open and striped bars, respectively). Cell culture supernatants were assayed for PGE_2_. (B–C) The expression level of IL-1R1 was determined by FACS analysis on monocytes (B) and macrophages (C) untreated (dashed lines) or following activation with LPS at 20 ng/ml overnight (solid lines); filled histograms in panels B and C show staining with IgG-PE isotype control antibody. The Delta Mean Fluorescence Intensity (ΔMFI) was calculated by subtracting the isotope control MFI from the experimental values. The IL-1R1 ΔMFI for untreated and LPS-activated cells was 13.2±6.4 and 73.2±8.1 for monocytes (B) and 13.0±3.3 and 5.2±5.5 for macrophages (C), respectively, n = 3. Histograms show results from one representative experiment, each measuring at least 10,000 events. (D–E) Monocytes were incubated with human rIL-1β at 0, 10 or 100 ng/ml in the absence or presence of Kineret for 18 h (open and solid bars, respectively). Cell culture supernatants were assayed for PGE_2_ (D) or for IL-6 (E). The data in A, D, and E are shown as means ± STDEV for triplicate wells in the PGE_2_ and IL-6 and assay. This is representative of three experiments with similar results, ***p≤0.001, **p≤0.01; ns, not significant.

Partial reduction of PGE_2_ production in monocytes treated with ZVAD suggested an IL-1β-dependent mechanism. We explored this further by treating monocytes and macrophages with LPS in the absence or presence of the IL-1R antagonist, Kineret. PGE_2_ production in both cell types was observed following LPS stimulation, but was partially reduced by Kineret (30.5±5.6%, n = 3) only in monocytes but not in macrophages suggesting that macrophages might not express IL-1R1 (Fig. 4A). Flow cytometry analysis found negligible levels of IL-1R1 on the surface of untreated monocytes and macrophages. However, activation with LPS induced a strong increase in IL-1R1 expression on monocytes but not on macrophages (Fig. 4 B and C dashed vs. solid lines). Based on these observations, it was expected that exogenous IL-1β would induce PGE_2_ in monocytes (but not in macrophages). Surprisingly, recombinant IL-1β (rIL-1β) did not induce PGE_2_ in either monocytes (Fig. 4D) or macrophages (data not shown). As a positive control for the biological activity of rIL-1β, significant amounts of IL-6 were measured in the same monocyte cultures in a dose-dependent manner, and rIL-1β-induced IL-6 production was blocked by Kineret (Fig. 4E).

**Figure 5:**
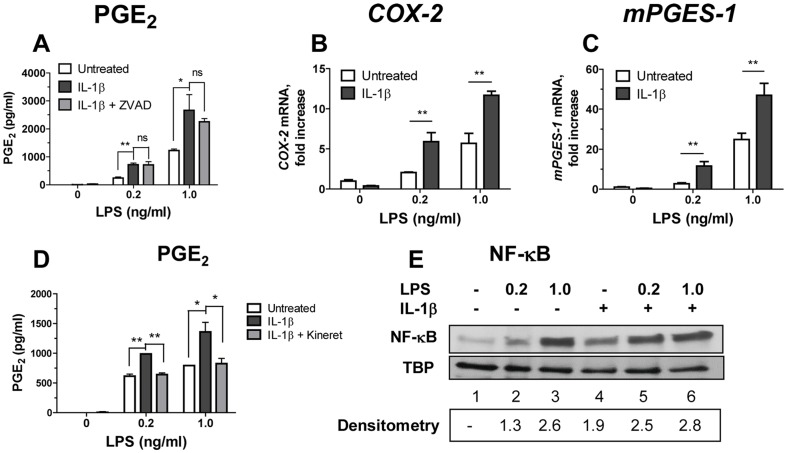
LPS priming is required for rIL-1β-induced *COX-2* and *mPGES-1* mRNA up-regulation and PGE_2_ production in monocytes. (A–D) Monocytes were left untreated or were primed with LPS for 1 h (open bars) or were primed with LPS followed by addition of rIL-1β at 100 ng/ml for 18 h (solid bars). In some cultures, LPS-primed monocytes were cultured with rIL-1β in presence of ZVAD (10 µg/ml, panel A, grey bars) or Kineret (100 µg/ml, panel D, grey bars). Cell culture supernatants and cells were collected and were assayed for PGE_2_ (A and D), or for *COX-2* and *mPGES-1* mRNA (B and C), respectively. The relative values of *COX-2* and *mPGES-1* mRNA expression were normalized using qPCR reactions with *β-actin* primers performed in the same samples. The data is shown as mean PGE_2_±STDEV for triplicate wells in the PGE_2_ assay and mean mRNA fold increases over untreated cells ± STDEV calculated for triplicate wells in A and D and in B and C, respectively, **p≤0.001; *p≤0.05; ns, not significant (p≥0.05). This experiment was performed with monocytes from three separate donors with similar results. The data is shown for one representative experiment. (E) Monocytes were left untreated (lane 1) or were treated with LPS at 0.2 or at 1.0 ng/ml alone (lanes 2 and 3) or with IL-1β at 100 ng/ml alone (lane 4) or with LPS at 0.2 and at 1.0 ng/ml and IL-1β together (lane 5 and 6). At 15 min post-treatment, nuclear extracts were obtained from collected cells and resolved in SDS-PAGE. NF-κB and TBP (control) were detected by Western Blotting. Densitometry values in lower panel represent fold increase in the intensity of NF-κB band in treated over untreated monocytes in this experiment (shown in lane 1). This experiment was performed twice with the same results; data are from one representative donor.

We next investigated whether LPS priming is required for IL-1β responsiveness. Monocytes were primed with low doses of LPS (0.2 and 1.0 ng/ml) for 1 h followed by addition of rIL-1β for 18 h (Fig. 5). In the absence of LPS priming (at 0 concentration of LPS in Fig. 5), monocytes activated with rIL-1β did not produce PGE_2_ or up-regulate *COX-2* and *mPGES-1* (Fig. 5 A–C). In contrast, PGE_2_ production as well as *COX-2* and *mPGES-1* expressions were significantly augmented by rIL-1β in LPS-primed monocytes, p≤0.01 (Fig. 5 A–C, solid bars). PGE_2_ production induced by exogenous rIL-1β in LPS-primed monocytes was not significantly reduced by ZVAD (Fig. 5A, shaded vs. solid bars), confirming that under these conditions, endogenously produced IL-1β was not as efficient as rIL-1β. On the other hand, IL-1R blocker, Kineret, abrogated the effect of exogenous rIL-1β (Fig. 5D, solid vs. shaded bars). In contrast to the findings in monocytes, PGE_2_ was not up-regulated by rIL-1β in LPS-primed macrophages in agreement with the lack of IL-1R1 up-regulation in LPS-treated macrophages (data not shown).

NF-κB plays a major role in transcription of *COX-2* gene (reviewed in [24]). Therefore, we investigated whether exogenous human rIL-1β increases activation of NF-κB in monocytes primed with LPS. To that end, nuclear extracts were prepared from monocytes treated for 15 minutes with LPS alone, with rIL-1β alone, or with LPS plus rIL-1β. These extracts were resolved in SDS-PAGE and probed in Western Blot with anti-NF-κB (p65) antibodies (Fig. 5E). Low levels of NF-κB were detected in the nuclei of untreated monocytes (lane 1). LPS at 0.2 and 1.0 ng/ml induced 1.3 and 2.6 fold increase in nuclear NF-κB, respectively (lanes 2 and 3), and rIL-1β alone induced 1.9 increase in nuclear NF-κB (lane 4). A combination of LPS at low concentration (0.2 ng/ml) with rIL-1β increased levels of nuclear NF-κB over LPS alone, however, this increase was less than additive (2.5 fold). At the same time, 1.0 ng LPS with rIL-1β did not induce further increase in the levels of nuclear NF-κB compared with LPS alone (2.8 vs. 2.6 fold increase, respectively) (Fig. 5E, lanes 3 vs. 6). These data confirmed that rIL-1β induced nuclear translocation of NF-κB in monocytes at levels sufficient to induce IL-6 (Fig. 4). However, the presence of activated NF-κB in LPS-primed monocytes that were treated with rIL-1β was not sufficient to induce up-regulation of *COX-2* mRNA and production of PGE_2_. Therefore, other LPS-induced signaling may be required for IL-1β induced PGE_2_ production.

### TLR2 agonists induce PGE_2_ production in human monocytes only through IL-1β independent mechanisms

**Figure 6:**
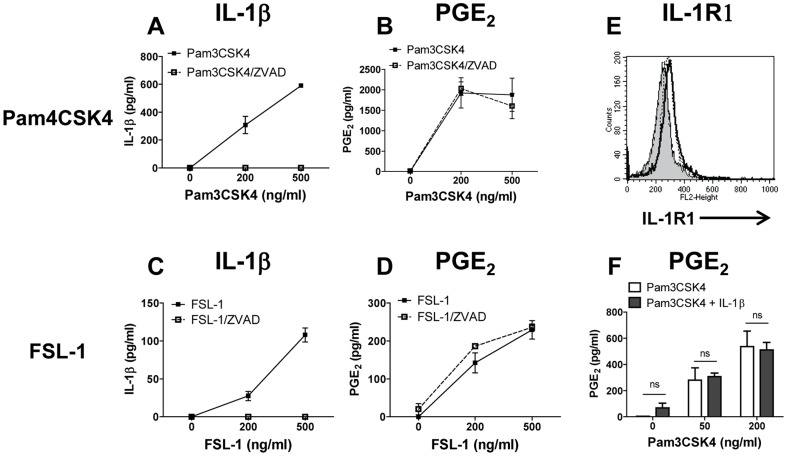
Caspase-1 and IL-1β do not play a role in TLR-2 ligand-induced PGE_2_ production in monocytes. Monocytes were treated with Pam3CSK4 (A, B) or with FSL-1 (C, D) with or without ZVAD for 18 h (broken and solid lines respectively). Cell culture supernatants were assayed for IL-1β (A, C) and for PGE_2_ (B, D). (E) IL-R1 surface expression was determined by FACS analysis on untreated monocytes (dashed line) or on monocytes following activation with Pam3CSK4 or with FSL-1 at 500 ng/ml overnight (solid and dotted lines, respectively); filled histogram shows staining of untreated monocytes with isotype control. IL-1R1 ΔMFI for untreated monocytes and for monocytes activated with Pam3CSK4 or with FSL-1 was 14.2±5.1, 55.5±10.5, and 47.3±8.4, respectively, n = 3. Histograms show results from one representative experiment, each measuring at least 10,000 events. (F) TLR2 agonist does not prime monocytes for rIL-1β response. Monocytes were primed with Pam3CSK4 for 1 h at indicated concentrations (open bars) or were primed with Pam3CSK4 followed by addition of rIL-1β at 100 ng/ml for 18 h (solid bars). Cell culture supernatants were collected and assayed for PGE_2_ protein. The data are shown as mean ± STDEV for PGE_2_ and IL-1β protein concentration calculated from triplicate wells in the PGE_2_ assay and in IL-1β ELISA, respectively. This is representative of three experiments; ns, not significant.

We recently reported that similar to the TLR4 agonist LPS, strong TLR2 agonists such as Pam3CSK4 and FSL-1 induced PGE_2_ and pro-inflammatory cytokines in human monocytes and in MM6 cells [14]. We therefore sought to determine whether production of PGE_2_ following activation of TLR2 is augmented by IL-1β. Pam3CSK4 and FSL-1 induced both IL-1β and PGE_2_ in human monocytes (Fig. 6 A, C and B, D, respectively). As expected, ZVAD completely blocked TLR2-induced IL-1β (Fig. 6 A and C, open squares). However, no effect of ZVAD on PGE_2_ production was noted (Fig. 6 B and D, open squares). These findings were confirmed by the lack of inhibitory effect of ZVAD on *COX-2* and *mPGES-1* mRNA up-regulation in monocytes treated with FSL-1 and Pam3CSK4 (data not shown).

To explore this difference between TLR4 and TLR2 agonists, we assayed the levels of IL-1R1 expression on Pam3CSK4- or on FSL-1-activated monocytes (Fig. 6E). IL-1R1 expression was increased in monocytes activated with Pam3CSK4 or with FSL-1compared with untreated monocytes (Fig. 6E dashed and dotted lines, respectively, vs. solid line), but not as strongly as was observed after LPS priming (Fig. 4B). Furthermore, in contrast to the findings with LPS-primed monocytes, PGE_2_ production in monocytes treated with 50 or 200 ng/ml of Pam3SCK4 did not increase when rIL-1β was added to the cell cultures (Fig. 6F, black vs. white columns). Similar results were obtained with FSL-1-primed monocytes (data not shown).

### Activation of IRF3 was required for IL-1β-induced up-regulation of PGE_2_ in LPS-primed monocytes

**Figure 7:**
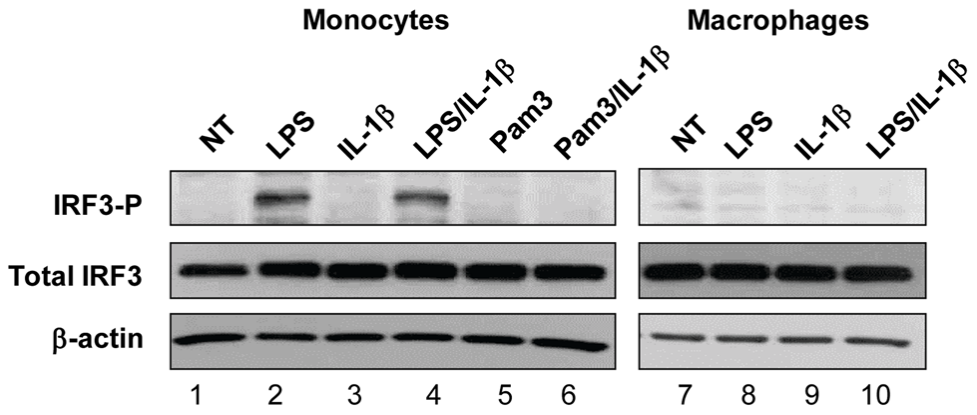
IRF3 is phosphorylated in monocytes but not in macrophages following activation with LPS. Monocytes (lanes 1–6) and differentiated macrophages (lanes 7–10) were left untreated (lanes 1 and 7) or were treated for 1 h with: LPS alone at 1 ng/ml (lanes 2, 8); Pam3CSK4 alone at 50 ng/ml (lane 5); with IL-1β alone at 100 ng/ml (lanes 3, 9), or with LPS + IL1β (lanes 4, 10); or with Pam3CSK4 + IL-1β (lane 6). Following incubation, cell lysates were prepared and resolved in SDS-PAGE. Phospho-IRF3, total IRF3, and β-actin were detected after Western Blotting. The experiment was performed 3 times with similar results.

Binding of LPS to TLR4 activates two signal transduction cascades; one through MyD88 and the other through TRIF/TRAM adaptor molecules, culminating in the nuclear translocation of NF-κB and of interferon regulatory factors (IRF) including IRF3, respectively [18]. Triggering of TLR2 by Pam3CSK4 initiates only MyD88-dependent pathway [18]. To determine whether the TRIF/IRF3 pathway plays a role in IL-1β-induced PGE_2_ induction in LPS-primed monocytes, we investigated whether IRF3 is phosphorylated in human monocytes and macrophages in response to TLR4 (LPS) vs. TLR2 (Pam3CSK4) agonists and whether IL-1β affected IRF3 phosphorylation in agonist-primed monocytes and macrophages. Monocytes and macrophages were cultured alone, with either LPS or Pam3CSK4, and with rIL-1β alone or in combination with LPS or Pam3CSK4 (Fig. 7). Cells were lysed and proteins were resolved by SDS-PAGE and probed in Western Blots with antibodies against phosphorylated IRF3 (Ser 396, IRF3-P) or against total IRF3. Total IRF3 was detected in all cells at similar levels irrespective of treatment (Fig. 7, middle panel). IRF3-P was detected in LPS-treated but not in Pam3CSK4-treated monocytes (Fig. 7 lane 2 vs. 5). Addition of rIL-1β alone did not induce phosphorylation of IRF3 (lane 3) and rIL-1β did not increase the levels of phosphorylated IRF3 in either LPS-treated (lane 4) or Pam3-treated monocytes (lane 6). These findings confirmed that IL-1β induces NF-kB translocation (Fig. 5E) but not IRF3 activation (Fig. 7). Importantly, we did not detect IRF3-P in LPS-, or in rIL-1β-, or LPS/rIL-1β-treated macrophages (Fig. 7, lines 8–10). These data suggested that monocytes and macrophages differ in signaling pathways initiated by TLR4: in monocytes but not in macrophages triggering of TLR4 induces phosphorylation of IRF3 suggesting activation of TRIF/TRAM signaling pathway.

### siRNA targeting for *TRIF*, *TRAM*, and *IRF3* reduce *COX-2* mRNA induction in LPS-primed IL-1β treated U373-CD14 cells

**Figure 8:**
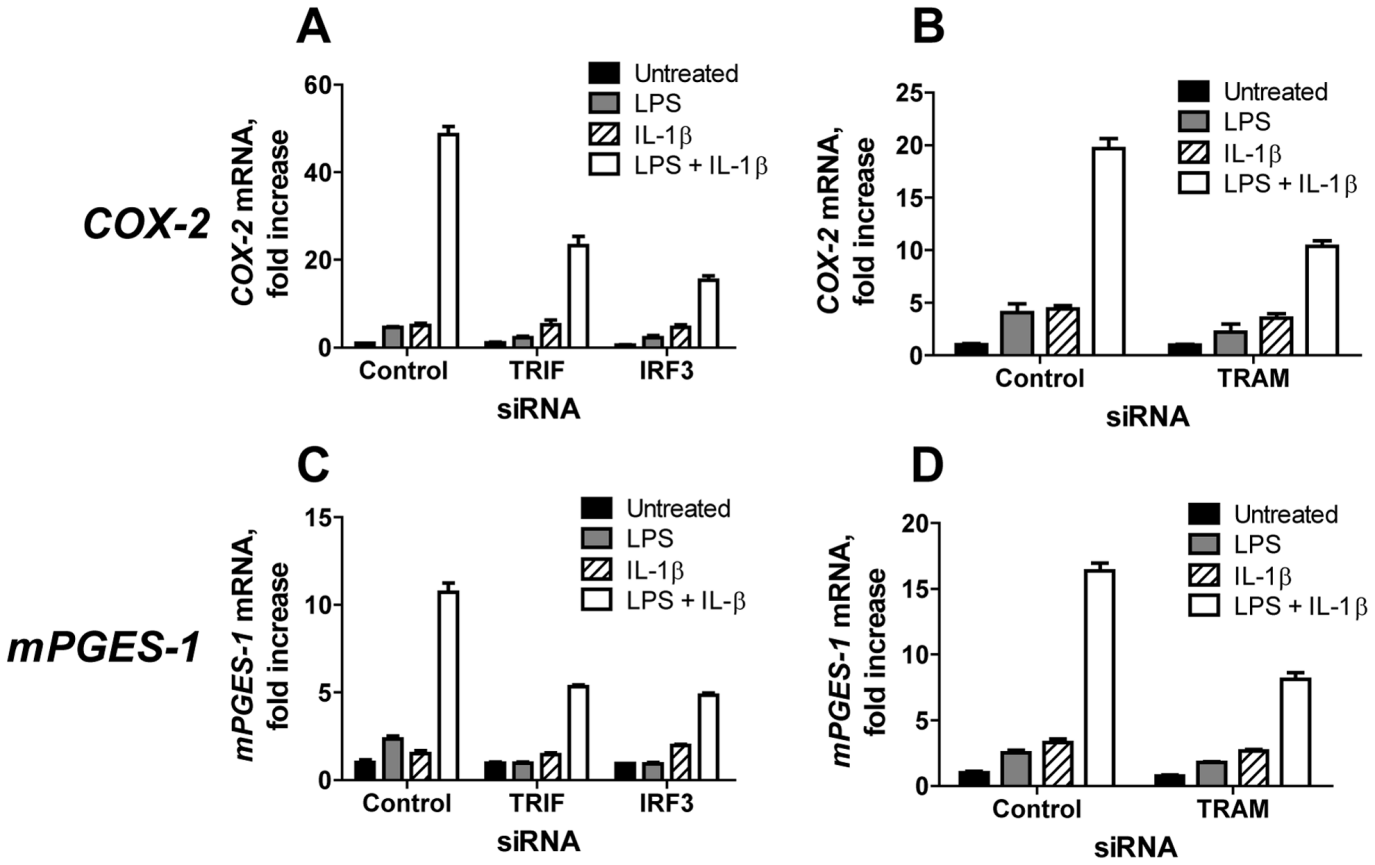
IRF3, TRIF, and TRAM siRNA reduced *COX-2* and *mPGES-1* in rIL-1β-treated LPS-primed U373-CD14 cells. U373-CD14 cells were transfected with control siRNA or with siRNA targeting *TRIF* or *IRF3* (A, C), or *TRAM* (B, D). Transfected cells were left untreated (solid bars), or were incubated with LPS at 1 ng/ml (shaded bars), with rIL-1β at 100 ng/ml for 18 h (striped bars), or were primed for 1 h with LPS and incubated with rIL-1β for additional 18 h (open bars). Cells were collected and *COX-2* mRNA (A, B) and *mPGES-1* mRNA (C, D) expressions were assayed by qPCR. The data are shown as fold increase over control PCR with primers specific to *β-actin*, run in the same samples. PCR was performed using triplicate wells and the data are shown as mean fold increase ± STDEV. This is representative of three experiments.

To further investigate the involvement of TRIF-TRAM signaling pathway in PGE_2_ production, we examined the effects of siRNA targeting *IRF3*, *TRIF*, and *TRAM* on PGE_2_ production in LPS-primed cells treated with rIL-1β. Because monocytes are difficult to transfect, we used U373-CD14 astrocytoma cells that resemble CNS macrophages [20]. Earlier studies have shown that in U373 cells, LPS induced nuclei translocation of IRF3 [25]. In agreement with these observations, we confirmed that IRF3-P was detected in U373-CD14 astrocytoma cells at 1 h post-treatment with LPS (Figure S1, lane 2). U373-CD14 cells do not produce PGE_2_ (data not shown) and therefore, we evaluated expression of *COX-2* and *mPGES-1* mRNA as a surrogate assay for PGE_2_. U373-CD14 cells were transfected with control siRNA or with *TRIF*, *IRF3*, or *TRAM* siRNA. The efficiency of gene silencing was assayed by performing qPCR and Western Blotting for the targeted genes and ranged between 75–80% (Figure S2). To follow *COX-2/mPGES-1* induction, transfected U373-CD14 cells were left untreated, or treated with LPS alone, rIL-1β alone, or were primed with LPS for 1 h followed by incubation with rIL-1β for 18 h (Fig. 8). In cells transfected with control siRNA, LPS and rIL-1β alone induced only small increases in *COX-2* mRNA expression compared with no treatment control, while, priming with LPS followed by incubation with rIL-1β induced a 20- to 50-fold increase in *COX-2* mRNA expression (Fig. 8, A and B, open bars). Transfection of U373-CD14 cells with siRNA targeting *TRIF*, *IRF3* (panel A) or *TRAM* (panel B) induced 2- and 3-fold reductions in the levels of *COX-2* mRNA in LPS/rIL-1β treated cells compared with cells transfected with control siRNA (Fig. 8 A, B, open bars). Similar reduction in *mPGES-1* mRNA expression was observed in LPS-primed rIL-1β-treated U373-CD14 transfected with siRNA targeting *TRIF*, *IRF3* or *TRAM* (Fig. 8, panels C and D, open bars). These data suggested that TRIF-TRAM-IRF3 signaling pathway downstream of TLR4 results in up-regulation of the key enzymes involved in PGE_2_ synthesis in response to IL-1β.

**Figure 9:**
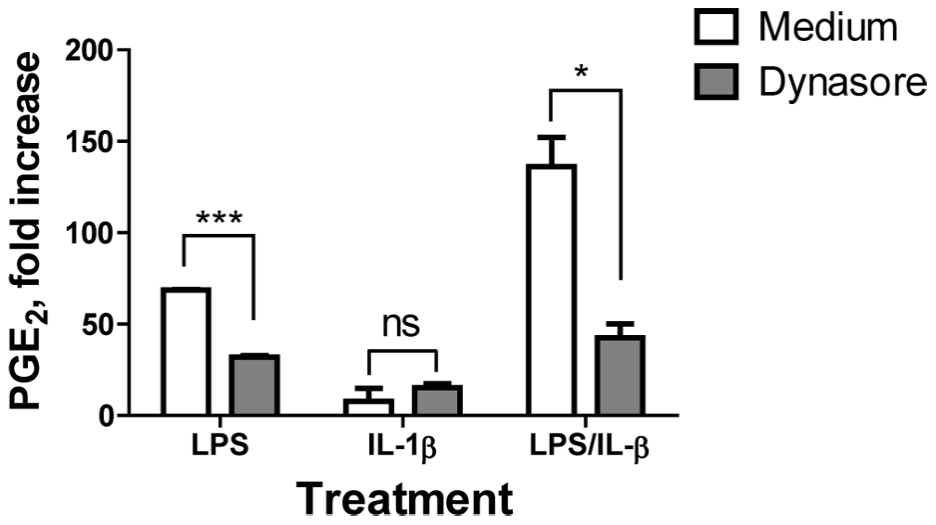
Endocytosis of TLR4 is required for enhancement of PGE_2_ production by IL-1β in LPS-primed monocytes. Monocytes were left untreated or were incubated with LPS alone (10 ng/ml), or with human rIL-1β alone (100 ng/ml) or were primed with LPS for 1 h followed by 18 h incubation with rIL-1β in presence of GTPase inhibitor dynasore (10 µM, grey bars) or medium alone (open bars). At the end of incubation period, cell culture supernatants were assayed for PGE_2_ production. Data are shown as fold increase in PGE_2_ over untreated monocytes. Data represent average ± STDEV for monocytes obtained from three donors. Asterisks denote significant differences between cells incubated in the absence or in presence of dynasore, ***p≤0.001; *p<0.05; ns, not significant (p>0.05).

LPS-induced internalization of TLR4 was shown to play an essential role in TRIF-TRAM recruitment [26]. To examine whether blocking of LPS-induced internalization of TLR4 is required for IL-1β-dependent increase in PGE_2_ production, monocytes were treated with dynasore, a specific inhibitor of GTPase dynamin, before priming with LPS and subsequent incubation with rIL-1β (Fig. 9). In the absence of dynasore, rIL-1β-induced two-fold increase in PGE_2_ production compared with LPS alone (open bars). Treatment with dynasore prior to LPS abrogated the effect of rIL-1β (grey bars). These data suggest that the ability of IL-1β to augment PGE_2_ production in LPS-primed monocytes requires TLR4 internalization.

**Figure 10:**
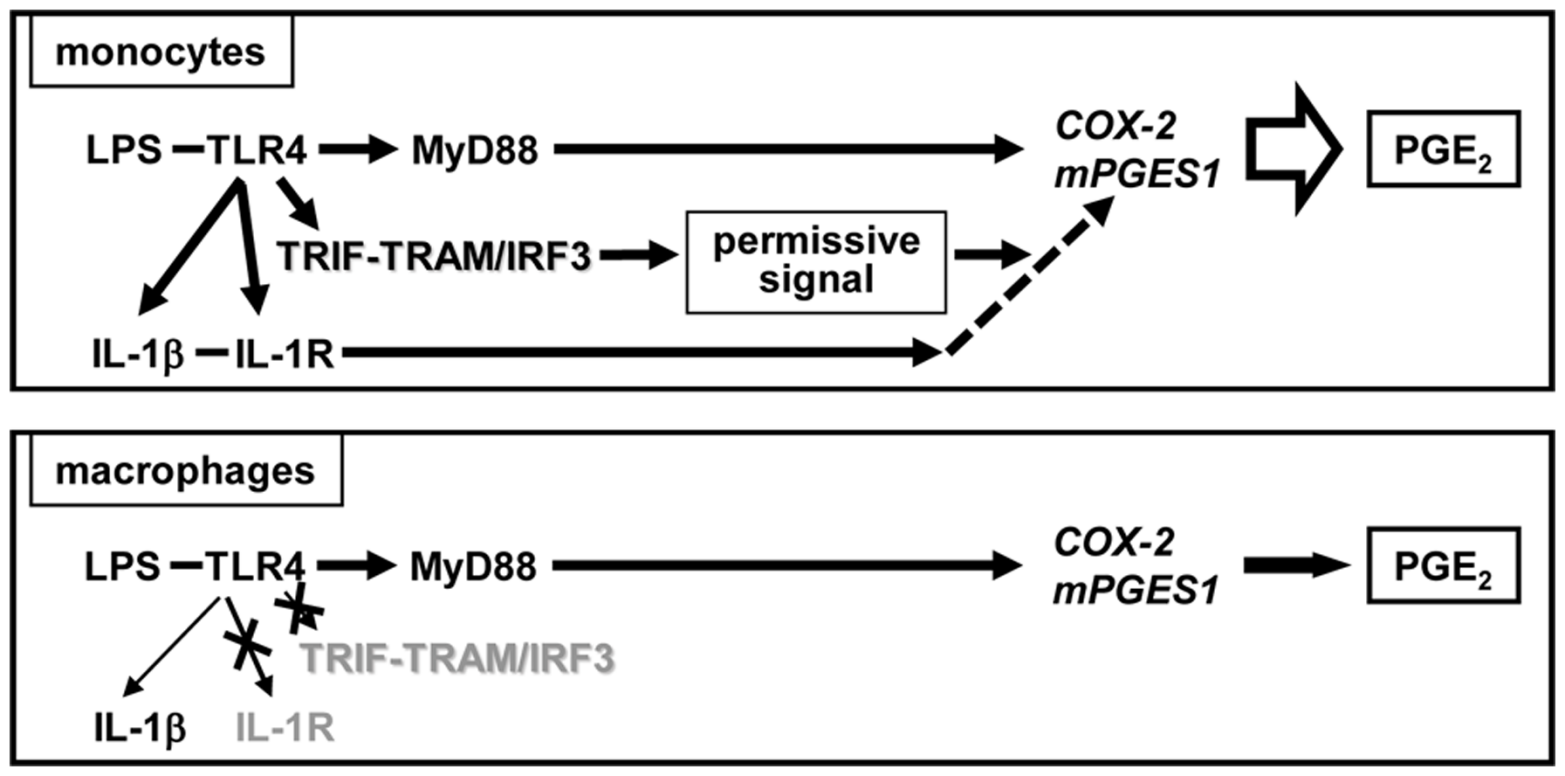
Model of IL-1β dependent and IL-1β independent pathways of PGE_2_ production in human monocytes and macrophages.

Together, these data demonstrated that monocytes and macrophages respond to TLR4 engagement via different signaling pathways that lead to COX-2 up-regulation and PGE_2_ production (Fig. 10). Unlike macrophages, in monocytes activated through TLR4, IL-1β contributes to PGE_2_ production. This PGE_2_-augmenting activity of IL-1β requires TLR4 internalization with subsequent signaling through TRIF/IRF3 pathway. The TLR2-induced PGE_2_ production in both cell types is not augmented by IL-1β.

## Discussion

Upon differentiation from the precursors in the bone marrow, monocytes enter the circulation where they patrol the blood vessel lumen and enter tissues at the site of infection to differentiate into tissue macrophages. Both monocytes and macrophages are innate immune cells that share the common duty of defending the host from invading pathogens. This function is achieved with the help of a battery of PRRs expressed on monocytes and macrophages that allows them to sense invading pathogens and endogenous danger signals. In this study we showed that although triggering of PRR on both monocytes and macrophages induces inflammatory molecules such as IL-1β and PGE_2_, the pathways leading to PGE_2_ production is different between these two cell types and between TLR4 and TLR2 ligands. The main findings are: (a) the TLR4 agonist LPS induced PGE_2_ in both monocytes and macrophages at different levels (monocytes >> macrophages); (b) in macrophages, PGE_2_ induction was entirely IL-1β-independent as evidenced by the lack of inhibition by caspase-1 inhibitor, ZVAD, and by the IL-1R antagonist, Kineret; (c) in contrast, in monocytes, PGE_2_ production was partially sensitive to ZVAD and to Kineret, indicating that IL-1β contributed to PGE_2_ production in LPS-treated monocytes; (d) unlike LPS, PGE_2_ production in FSL-1- or Pam3CSK4-activated monocytes was not affected by ZVAD and therefore IL-1β-independent; (e) IL-1R was up-regulated on monocytes (but not macrophages) following priming with TLR4 agonist and to a lesser degree by TLR2 agonists yet TLR4 but not TLR2 agonists could prime monocytes for IL-1β-augmented PGE_2_ production; (f) siRNA targeting TRIF, TRAM, and IRF3 prevented the IL-1β augmenting effect on *COX-2*/*mPGES-1* expression in LPS-primed U373-CD14 cells; (f) IRF3-P was detected in LPS- but not Pam3CSK4-treated monocytes. No IRF3 phosphorylation was seen in LPS-treated macrophages.

Based on these findings we suggest that in macrophages, signaling through TLR4/MyD88 pathway is sufficient to induce up-regulation of *COX-2* and *mPGES-1* transcription with subsequent PGE_2_ production (Fig. 10). In monocytes, LPS induces PGE_2_ by two pathways: the IL-1β-independent pathway similar to the one in macrophages that involves direct up-regulation of *COX-2* and *mPGES-1* through MyD88. In addition, LPS up-regulates expression of IL-1R1 on monocytes and primes monocytes to the augmenting effect of IL-1β that requires LPS-induced internalization of TLR4 with subsequent signaling through TRIF/TRAM adaptor molecules and activation of IRF3 (IL-1β-dependent mechanism of PGE_2_ production) (Fig. 10). *In vivo*, enhanced PGE_2_ production in monocytes might be triggered by endogenous IL-1β produced by the same cell after TLR4 activation, or can be provided in-trans from bystander cells. The combined activation pathways results in higher levels of PGE_2_ production in activated (inflammatory) monocytes compared with macrophages and may explain the rapid fever following TLR4 triggering.

Human elutriated monocytes and GM-CSF differentiated macrophages, which are considered to resemble inflammatory (M1) type of tissue macrophages [27], were used in the current study. A dramatic difference in the levels of IL-1β produced by these two cell types was observed, at 5 and at 50 ng/ml of LPS, there was a 30- and 14-fold higher quantity of IL-1β released by monocytes compared with macrophages. The same cell cultures that were assayed for IL-1β were tested for the presence of PGE_2_ and although there was a dramatic difference in IL-1β between the two cell types, the levels of PGE_2_ were only 4-6 times lower in macrophages than in monocytes. Therefore, there was no direct correlation between IL-1β and PGE_2_ production in monocytes and macrophages in response to TLR4 activation. Moreover, following LPS activation, a pan-caspase inhibitor ZVAD blocked IL-1β production in both cell types, while it had no effect on PGE_2_ produced in macrophages. These data are in agreement with previous reports showing that LPS-activated monocytes produce much higher quantities of IL-1β than differentiated macrophages [28], [29]. This difference was considered to be the outcome of the presence of constitutively activated caspase-1in monocytes but not in macrophages [28] and the need for second signal such as silica crystals or alum to induce inflammasome activation with subsequent release of mature IL-1β [30]. Interestingly, recent study by Barbera-Cremades et al have shown that LPS-primed human and mouse macrophages produced PGE_2_ only in presence of exogenous adenosine triphosphate (ATP) and under these conditions, PGE_2_ production was P2X7 receptor dependent [31]. In our studies, PGE_2_ and IL-1β were detected in human GM-CSF-derived macrophages activated with LPS in the absence of exogenous ATP, and a selective antagonist of P2X7 receptor (A438079) had no effect on LPS-alone induced PGE_2_ (data not shown). In spite of these differences, the important conclusion of our study and that of Barbera-Cremades et al is that PGE_2_ production by macrophages activated by LPS is caspase-1-independent suggesting that unlike IL-1β, induction of PGE_2_ by LPS in macrophages most likely does not require inflammasome activation [31]. This conclusion is also supported by the data in gene knock-out mice where absence of major components of NLRP3 inflammasome complex did not abrogate silica and alum-induced PGE_2_ in LPS-primed macrophages [30].

In monocytes, the effect of caspase-1 inhibitor on PGE_2_ was different from macrophages: our experiments showed that caspase-1 inhibitor and also IL-1R inhibitor partially reduced LPS-induced PGE_2_, suggesting that unlike macrophages, in monocytes LPS-induced PGE_2_ might be partially depended on IL-1β and/or caspase-1. Processing of bioactive IL-18 also depends on the function of caspase-1 and therefore is sensitive to ZVAD. However, IL-18 does not induce *COX-2* up-regulation *in vitro* [32] and does not cause fever in animals [33], therefore we did not investigate the role of IL-18 in inducing PGE_2_ in monocytes/macrophages.

Previous studies showed that a direct exposure of human fibroblasts, synovial fibroblasts, or of chondrocytes to rIL-1β-induced PGE_2_ [34]–[36]. However in our experiments, human rIL-1β did not induce PGE_2_ in human monocytes, differentiated macrophages, and in total PBMCs derived from multiple donors (data not shown) suggesting that the ability of rIL-1β to directly induce PGE_2_ is cell type specific or that some critical signaling pathways responsible for induction of the COX-2 gene transcription in response to IL-1β are absent in monocytic cells. In our experiments, rIL-1β induced nuclear translocation of NF-κB and production of IL-6 in monocytes confirming that IL-1β induced one of the major transcription factors involved in *COX-2* gene transcription. These data also confirmed that signaling from IL-1R1 in monocytes is not affected by ST2 negative regulator that has been shown to interfere with TLR4- or IL-1R-induced NF-κB activation [37] and suggested that similar to IL-1β, IL-6 alone does not induce PGE_2_ in monocytes.

These data indicated that although rIL-1β by itself failed to induce PGE_2_ in monocytes, endogenously produced IL-1β could support up-regulation of PGE_2_ in monocytes but only if they were activated (primed) through TLR4. This hypothesis was confirmed by experiments showing that LPS-primed monocytes up-regulated surface IL-1R1 and increased *COX-2* and *mPGES-1* expression and PGE_2_ protein production in response to rIL-1β. Unlike monocytes, LPS-activated macrophages did not up-regulate surface IL-1R1 and did not increase PGE_2_ production in response to exogenous rIL-1β in agreement with recently published data in mice [30]. These data suggested that responsiveness to IL-1β might be solely linked to LPS-induced up-regulation of IL-1R1 in monocytes but not in macrophages. To probe this question further, we compared priming of monocytes with LPS vs. TLR2 agonists and found that TLR2 agonists also up-regulated IL-1R1 in monocytes although to a lesser degree than LPS, yet TLR2 agonist did not prime monocytes for increased PGE_2_ secretion in response to exogenous rIL-1β. It is unlikely that the smaller increase in IL-1R1 in Pam3CSK4- or in FSL-1-treated monocytes compared with LPS-treated monocytes is the sole explanation for the lack of priming by TLR2 agonists since very few surface IL-1R1 molecules are sufficient for signal transduction [38]. Rather, our data suggested that LPS induced additional signals in monocytes (but not in differentiated macrophages) that were required to up-regulate *COX-2/mPGES-1* gene transcription with subsequent PGE_2_ production in response to rIL-1β.

The inability of TLR2 ligand**s** to prime monocytes for response to rIL-1β suggested that the permissive signals derived from TLR4 might be transmitted through the TLR4/TRIF-TRAM/IRF3 pathway. This possibility was confirmed in knock-down experiments, where siRNA coding for *TRIF*, *TRAM*, or for *IRF3* abrogated *COX-2* mRNA up-regulation in response to exogenous rIL-1β in LPS-primed U373-CD14 cells. Together with the evidence of LPS- (but not TLR2 ligand) induced IRF3 phosphorylation in monocytes and inhibition of PGE_2_ production by blocking of TLR4 internalization our data suggested that LPS-induced activation of TRIF/IRF3 signaling pathway might play an important role in altering the ability of monocytes to respond to IL-1β. Interestingly, recent study by Zanoni et al in mice have shown the important role of tyrosine kinase Syk/PLCγ2 downstream of CD14 receptor that facilitates transport of CD14/TLR4 complex to endosomes with a subsequent activation of TRAM-TRIF cascade [39]. The role of Syk in our system was not investigated but it is intriguing to suggest that the ability of LPS but not of TLR2 ligands to prime monocytes for responsiveness to IL-1β may be augmented by CD14 as a receptor to LPS, especially since low doses of LPS were used to prime the human monocytes in our system. The nature of the IRF3-dependent permissive signal required for augmenting of PGE_2_ production by IL-1β is still under investigation. Although IRF3 was shown to play an essential role in the LPS-induced IFN-β gene expression in the mouse system [40], in our experiments, LPS alone or LPS with IL-1β did not induce IFN-β in monocytes and did not up-regulate CXCL10, a signature IFN-β induced genes (data not shown) [41]. Transcriptional regulation of the *COX-2* gene is very complex and may involve numerous signaling pathways depending on the specific stimulus and cell type including NF-kB, C/EBP, CREB, AP1, and PPARγ (reviewed in [24]). Studies are underway to determine whether TRIF/TRAM activation by TLR4 required for increased IL-1β-induced transcription of *COX-2* involves post-translational modifications of NF-κB, or is required for sustained NF-κB activation [42] or recruitment of other DNA binding proteins such as CREB binding protein (CBP) co-activator that has been shown to associate with IRF3 and play a critical role in transcription of *COX-2* [43].

The role of IL-1β as a primary pyrogenic cytokine is very well established. However, studies in animal models showed that TLR-induced fever can be cytokine independent (reviewed in [44]). For example, systemic injection of LPS resulted in similar elevations in body temperature in IL-1β-deficient and in wild type mice. Additionally, antibodies against IL-1β only partially reduced LPS-induced fever in rats [45], [46]. At the same time, IL-1β deficient mice were completely resistant to fever induced by subcutaneous injection of a non-microbial product turpentine in the model of local inflammatory response [47]. Thus our data contribute to these earlier observations by showing that PGE_2_ may be directly induced by TLR ligands in both human macrophages and monocytes, and this induction does not require IL-1β. At the same time, IL-1β can contribute to PGE_2_ production during local inflammation after recruitment of inflammatory monocytes. Our studies may also suggest different contributions by tissue macrophages and inflammatory monocytes to the rapid pyrogenic response to pathogens as well as to certain products (such as vaccine adjuvants) designed to jump-start the immune system by activating various PPRs/TLRs.

## Supporting Information

Figure S1 **IRF-3 is phosphorylated in U373-CD14 astrocytoma cells following activation with LPS.** Cell lysates were prepared from U373-CD14 cells untreated (NT, lane 1) or U373-CD14 cells incubated with 10 ng/ml of LPS for 1 h (lane 2) and were resolved in SDS-PAGE. Phospho-IRF3, total IRF3, and β-actin were detected after Western Blotting. The experiment was performed 3 times with similar results.
(TIF)

Figure S2 **Efficiency of transfection of U373-CD14 cells with IRF3, TRIF, or TRAM siRNA.** U373-CD14 cells were transfected with control siRNA or with siRNA targeting *IRF3*, *TRIF*, or *TRAM* and the levels of endogenous *IRF3, TRIF, and TRAM* mRNA expressions were assayed by qPCR (upper panels in A, B, and C, respectively). The data was normalized using PCR with primers specific to *β-actin* run in the same samples and is shown as fold difference compared to cells transfected with control siRNA. The data is shown as mean fold increase ± STDEV for triplicate wells. Cell lysates were prepared from cells transfected with control (C) or with siRNA targeting *IRF3*, *TRIF*, or *TRAM*, were resolved in SDS-PAGE and IRF3, TRIF, TRAM, and β-actin protein expressions were detected after Western Blotting (WB, lower panels in A, B, and C, respectively). The experiment was performed 3 times with similar results.
(TIF)
