# Supplementary figures and images for: Differences in PGE2 Production between Primary Human Monocytes and Differentiated Macrophages: Role of IL-1β and TRIF/IRF3
Source: PLoS One. 2014 May 28;9(5):e98517. doi: 10.1371/journal.pone.0098517 (PMC4037220; doi:10.1371/journal.pone.0098517)

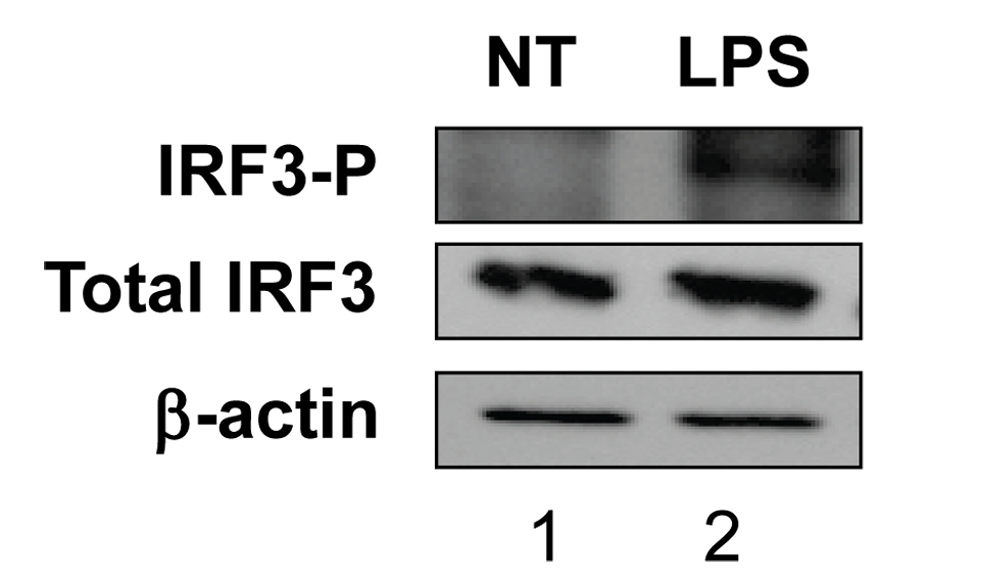

Supplement: Figure S1 — IRF-3 is phosphorylated in U373-CD14 astrocytoma cells following activation with LPS. Cell lysates were prepared from U373-CD14 cells untreated (NT, lane 1) or U373-CD14 cells incubated with 10 ng/ml of LPS for 1 h (lane 2) and were resolved in SDS-PAGE. Phospho-IRF3, total IRF3, and β-actin were detected after Western Blotting. The experiment was performed 3 times with similar results. (TIF) [file pone.0098517.s001.tif]

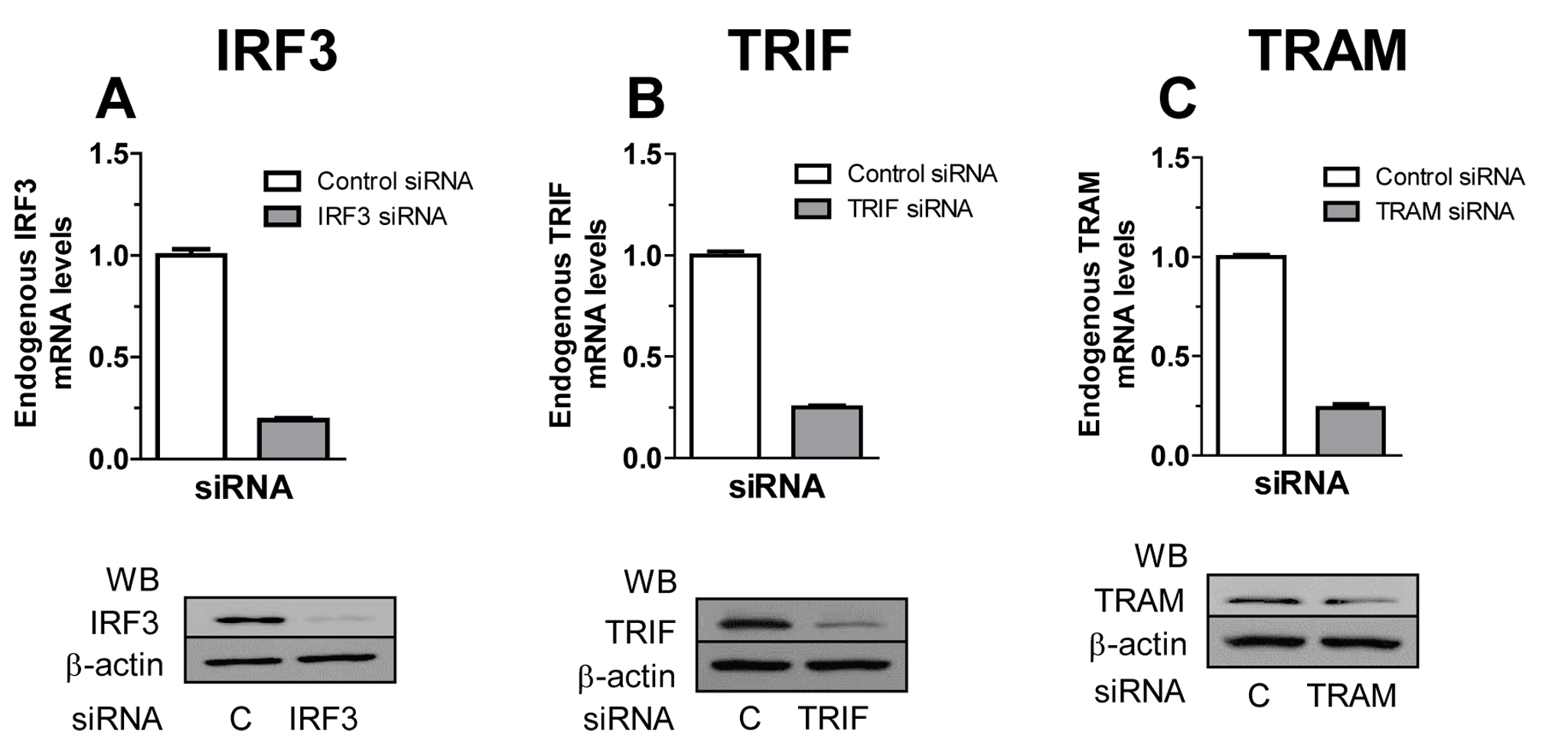

Supplement: Figure S2 — Efficiency of transfection of U373-CD14 cells with IRF3, TRIF, or TRAM siRNA. U373-CD14 cells were transfected with control siRNA or with siRNA targeting IRF3, TRIF, or TRAM and the levels of endogenous IRF3, TRIF, and TRAM mRNA expressions were assayed by qPCR (upper panels in A, B, and C, respectively). The data was normalized using PCR with primers specific to β-actin run in the same samples and is shown as fold difference compared to cells transfected with control siRNA. The data is shown as mean fold increase ± STDEV for triplicate wells. Cell lysates were prepared from cells transfected with control (C) or with siRNA targeting IRF3, TRIF, or TRAM, were resolved in SDS-PAGE and IRF3, TRIF, TRAM, and β-actin protein expressions were detected after Western Blotting (WB, lower panels in A, B, and C, respectively). The experiment was performed 3 times with similar results. (TIF) [file pone.0098517.s002.tif]
